# Diagnostic and Therapeutic Strategies in Evans Syndrome: A Case Report and Literature Review

**DOI:** 10.7759/cureus.64866

**Published:** 2024-07-18

**Authors:** Aadi R Palvia, Abhiram Rao Damera, Shikha Magar, Akshay Rahul Nandi, Mayank Goyal

**Affiliations:** 1 Internal Medicine, Kharghar Medicity Hospital, Navi Mumbai, IND; 2 Internal Medicine, MediCiti Institute of Medical Sciences, Hyderabad, IND; 3 General Medicine, Kempegowda Institute of Medical Sciences, Bengaluru, IND; 4 Internal Medicine, Dr. B.R. Ambedkar Medical College & Hospital, Bengaluru, IND; 5 Internal Medicine, Mayo Clinic, Rochester, USA

**Keywords:** direct anti-globulin test, intravenous immunoglobulins (ivig), prednisolone acetate, immune-mediated hemolysis, immune thrombocytopenia purpura, coombs positive hemolysis, acute pericardial effusion, immune thrombocytopenia (itp), autoimmune hemolytic anemia (aiha), evans’ syndrome

## Abstract

Evans syndrome (ES) is characterized by a combination of autoimmune hemolytic anemia (AIHA) and immune thrombocytopenia (ITP). Immune dysregulation, which results in the development of antibodies against blood cells, is its defining feature. ES being a diagnosis of exclusion requires a thorough workup to rule out other probable illnesses like lymphoproliferative diseases and systemic lupus erythematosus (SLE). We present the case of a 38-year-old male who experienced shortness of breath, chest discomfort, and generalized weakness. His medical history included recurrent anemia, thrombocytopenia, and pulmonary tuberculosis in remission. Hemolysis, thrombocytopenia, and a large pericardial effusion were discovered during the physical examination and investigations. An initial treatment strategy that included pericardiocentesis was performed. In combination with AIHA and ITP, the clinical and laboratory findings strongly suggested ES, which improved with prednisolone therapy. First-line treatments consist of corticosteroids and intravenous immunoglobulin; refractory cases may also require rituximab, thrombopoietin receptor antagonists, and sirolimus. Achieving remission and lowering relapse rates need careful patient monitoring and customized treatment programs.

## Introduction

Evans syndrome (ES), a rare eponymous condition first described in 1951 by Robert Evans et al. [1,2], is defined as the concomitant or sequential association of warm autoimmune hemolytic anemia (AIHA) with immune thrombocytopenia (ITP) and, less frequently, autoimmune neutropenia [3]. It can be primary or secondary to various conditions like autoimmune lymphoproliferative syndrome, systemic lupus erythematosus (SLE), autoimmune hepatitis, and hematological malignancies such as chronic lymphocytic leukemia [4]. ES usually has a chronic course marked by several relapses [5]. The exact pathophysiology of ES is not fully understood, but it has been observed that there is immune dysregulation with subsequent production of antibodies targeting the erythrocytes, platelets, and neutrophils [2]. One theory is that the production of interleukin-10 and interferon-gamma causes the activation of autoreactive antibody-producing B cells [6]. The clinical presentations of ES vary and are typically related to low RBC count and hemolysis, such as jaundice, dark urine, pallor, weakness, fatigue, and shortness of breath (SOB); or to thrombocytopenia, such as easy bruising, petechiae, mucocutaneous bleeds, epistaxis, and heavy menstrual bleeding; or low neutrophil counts, such as fever and increased infections [7]. Symptoms are similar to those of leukemia and lymphoma. A physical examination may reveal lymphadenopathy, hepatomegaly, and/or splenomegaly [6]. Most commonly, patients present with bleeding events; however, they may rarely present with thrombosis [8]. ES is a diagnosis of exclusion and requires a high index of suspicion. The direct antiglobulin test (DAT) is invariably positive [1]. Diagnosis may be delayed due to the appearance of a second cytopenia only months to years after the first [6]. In cases of anemia associated with reticulocytosis, elevated lactate dehydrogenase (LDH), low haptoglobin, and elevated indirect bilirubin, AIHA should be suspected [3]. Diseases causing cytopenia, such as SLE, thrombotic thrombocytopenic purpura, hemolytic uremic syndrome (HUS), and viral infectious diseases like HIV, should be excluded [1,6]. Other differentials include myelodysplastic syndromes, paroxysmal nocturnal hemoglobinuria, and hemolysis, elevated liver enzymes and low platelets syndrome in pregnancy [7]. Therapeutic options should be individualized based on the patient’s age, history, clinical presentation, severity, hemoglobin, and platelet counts. First-line therapies are glucocorticoids and intravenous immunoglobulin (IVIG). Other treatment options include immunosuppressants (cyclosporine, mycophenolate mofetil, vincristine, and azathioprine), rituximab, splenectomy, thrombopoietin receptor agonists, sirolimus, danazol, blood transfusion, and hematopoietic stem cell transplant [2-6].

## Case presentation

A 38-year-old male presented to the emergency department complaining of SOB, retrosternal chest discomfort, and generalized weakness. The symptoms had started gradually three days prior and had been worsening since then. Lying flat exacerbated the SOB and chest pain, while sitting provided relief. The patient also reported experiencing dark-colored stools, nausea, and generalized abdominal pain over the preceding two days.

The patient’s previous medical records revealed a history of pulmonary tuberculosis (TB), which had been effectively treated with antitubercular medications, resulting in remission. Complicating his medical background were repeated episodes of anemia and thrombocytopenia, which manifested both independently and concurrently.

**Figure 1:**
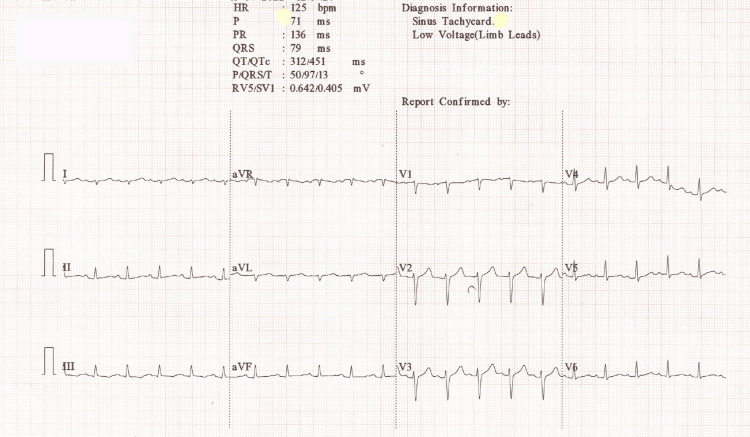
ECG findings suggestive of sinus tachycardia and low voltage in limb leads

On physical examination, the patient appeared pale with cold extremities. His vital signs revealed a pulse of 124 beats per minute, a blood pressure of 100/60 mmHg, a respiratory rate of 30 breaths per minute, and an oxygen saturation of 97%. Pallor and icterus were noted. Cardiovascular examination showed sinus tachycardia with muffled S1 and S2 heart sounds. Reduced breath sounds were auscultated at the base of the right lung during the respiratory examination. An abdominal examination revealed a mildly enlarged spleen and widespread discomfort. The ECG demonstrated low voltage in the limb leads and sinus tachycardia, as depicted in Figure 1.

**Figure 2:**
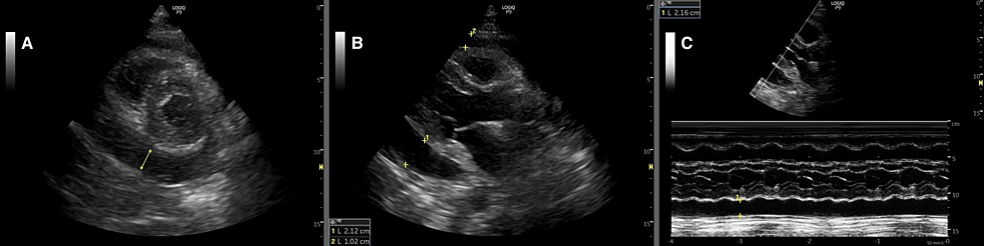
Two-dimensional echocardiogram showing features of pericardial effusion: (A) parasternal short axis view at the level of the left ventricle; (B) parasternal long axis view; and (C) parasternal long axis view motion mode at the level of the left ventricle

A large pericardial effusion was identified on a two-dimensional echocardiogram (Figure 2), necessitating an urgent pericardiocentesis. During the procedure, 500 mL of fluid was aspirated, and a pericardial catheter was placed. Post-procedure, the patient’s symptoms were relieved, and his vital signs stabilized.

A chest X-ray revealed haziness and atelectatic strands in the right lower zone with obliteration of the right costophrenic angle, consistent with mild pleural effusion. Abdominal ultrasound demonstrated an enlarged spleen measuring 13.4 cm in size, with increased parenchymal echotexture, indicative of grade 1 splenomegaly.

As shown in Table 1, the patient exhibited multiple hematological abnormalities. These included low levels of hemoglobin and haptoglobin, elevated LDH, an increased reticulocyte count, positive DAT, and elevated unconjugated bilirubin, all indicative of hemolysis. Iron studies, including ferritin, transferrin, and total iron-binding capacity, were within normal limits.

**Table 1:** Laboratory findings at the time of admission

Test	Result	Normal range	Unit
Hemoglobin	7.3	13.0-17.0	gm/dL
Red blood cells	2.81	4.0-6.0	million/mm^3^
Mean corpuscular volume	117.14	78-100	fL
Mean corpuscular hemoglobin	28.18	27-32	pg
Mean corpuscular hemoglobin concentration	34.31	30-36	g/dL
Red cell distribution width	12.9	10-16	%
White blood cells	4,500	4,000-11,000	mm^3^
Platelet count	78,000	150,000-450,000	mm^3^
Erythrocyte sedimentation rate	85	0-20	mm
C-reactive protein	85.1	0-6	mg/L
Haptoglobin	<30	30-200	mg/dL
Reticulocyte count	17	0.5-2.5	%
Lactate dehydrogenase	5,500	40-280	U/L
Serum iron	156	33-193	mcg/dL
Ferritin	404	30-400	ng/mL
Total iron-binding capacity	259	250-450	mcg/dL
Transferrin saturation	18	14-50	%
Total bilirubin	5.5	0.2-1.2	mg/dL
Direct bilirubin	0.4	0.10-0.40	mg/dL
Indirect bilirubin	5.1	0.10-1.00	mg/dL
Aspartate transaminase	59.4	5-40	IU/L
Alanine transaminase	42.1	5-40	IU/L
Alkaline phosphatase	108	40-125	IU/L
Serum creatinine	0.9	0.5-1.5	mg/dL
Blood urea nitrogen	10	8-21	mg/dL

Schistocytes, normocytic normochromic anemia, and immature RBCs consistent with hemolysis were identified in the peripheral blood smear (PBS). Thrombocytopenia further complicated the clinical picture. Additionally, occult blood was detected in stools, and blood was found in urinalysis, adding to the complexity of the case. To investigate potential causes of occult gastrointestinal bleeding, an esophagogastroduodenoscopy and a colonoscopy were conducted, both yielding unremarkable results.

At first, the patient’s history of pulmonary TB was considered a potential cause of the pericardial effusion. However, acid-fast bacilli were not detected in the pericardial fluid upon testing. TB was further ruled out by negative findings on the chest X-ray, mycobacterium TB culture, and interferon-gamma release assay. Additionally, tests for hepatitis B, hepatitis C, and HIV were negative.

The combination of AIHA and ITP is suggested by the patient’s history of recurrent anemia and thrombocytopenia, current symptoms, laboratory findings, including positive DAT, and the presence of schistocytes on PBS. When AIHA and ITP coexist without other plausible explanations, ES is strongly considered.

A trial of prednisolone was initiated to assess its effect on the patient’s condition. He responded remarkably well to the therapy, as evidenced by Table 2, depicting his laboratory findings five days after starting treatment. His significant improvement in symptoms following corticosteroid therapy aligns with the effective management typically seen in autoimmune diseases such as ES.

**Table 2:** Laboratory findings five days following the start of treatment

Test	Result	Normal range	Unit
Hemoglobin	10.4	13.0-17.0	gm/dL
Red blood cells	3.34	4.0-6.0	million/mm^3^
Red cell distribution width	13.2	10-16	%
White blood cells	4,800	4,000-11,000	mm^3^
Platelet count	130,000	150,000 to 450,000	mm^3^
Erythrocyte sedimentation rate	63	0-20	mm
C-reactive protein	58	0-6	mg/L
Reticulocyte count	12	0.5-2.5	%
Lactate dehydrogenase	780	40-280	U/L
Total bilirubin	2.5	0.2-1.2	mg/dL
Direct bilirubin	0.4	0.10-0.40	mg/dL
Indirect bilirubin	2.1	0.10-1.00	mg/dL
Aspartate transaminase	44	5-40	IU/L
Alanine transaminase	37	5-40	IU/L
Alkaline phosphatase	102	40-125	IU/L

## Discussion

ES is identified by ruling out other possible causes, such as HUS and disseminated intravascular coagulation, and combining a number of distinctive symptoms, such as ITP and AIHA. Hematologic evaluations, immunohematological procedures such as the DAT, and comprehensive serological analyses for autoantibodies are all part of the diagnostic criteria [7]. ES diagnosis requires a high index of suspicion and meticulously rules out other illnesses that can cause thrombocytopenia and hemolytic anemia. SLE, thrombotic thrombocytopenic purpura, marrow dysplastic syndromes, lymphoproliferative disorders, and viral infections must all be ruled out as part of a thorough differential diagnosis. Improved patient outcomes and proper therapy are made possible by an accurate diagnosis [1]. ES is usually diagnosed by PBS examination, a complete blood count, and confirmation of a positive DAT [9].

The diagnostic workup in our case indicated low hemoglobin, increased LDH, and low haptoglobin levels, indicating hemolysis, along with a history of recurrent anemia and thrombocytopenia. A positive DAT and the discovery of schistocytes on a PBS, along with these results, suggested ES.

The goals of therapy are to manage cytopenia relapses and achieve an overall response while minimizing complications. Depending on the severity and resistance of the disease, first-line therapy usually includes corticosteroids and IVIG, which are sometimes coupled with rituximab, splenectomy, or cytotoxic immunosuppression. Eltrombopag and romiplostim, which are agonists of the thrombopoietin receptor, are used to treat thrombocytopenia; in certain situations, plasma exchange (PEX) may be necessary. Hematologic parameters and hemolysis indicators are used to assess treatment results, with the goal of achieving durable remission and a decrease in relapse rates across multiple lines of therapy [9].

In our case, the administration of prednisolone led to a notable improvement in laboratory markers and a considerable improvement in clinical status. This outcome is consistent with the standard therapeutic strategies for autoimmune diseases such as ES.

Severe infections and thromboembolic events are among the serious risks and complications associated with ES. In order to improve patient outcomes and provide efficient long-term treatment, these challenges must be managed. Numerous investigations have examined various aspects of ES, such as available treatments and potential hazards.

According to studies, individuals with ES, especially those with warm AIHA, have a higher risk of venous thromboembolism (VTE), which includes pulmonary embolism and deep vein thrombosis. Even in the absence of active thrombocytopenia, a defining feature of ITP, this elevated risk endures. Improving patient outcomes requires early detection and adequate care of thromboembolic events in ES due to their potential severity. In order to reduce this elevated risk, physicians should keep a high index of suspicion for VTE in patients with ES, making sure that diagnostic evaluations are completed promptly and that preventive or therapeutic treatments are started [10].

ES presents significant healthcare issues, especially when co-occurring TB complicates the situation. Usually presented with warm AIHA and ITP, ES requires immunosuppressive therapy (rituximab, cyclophosphamide, and glucocorticoids) to induce remission. However, using these drugs makes one more vulnerable to infections like TB, which can make treatment plans more difficult. When TB is discovered accidentally in ES patients, antitubercular medication must be started immediately, coupled with cautious immunosuppression, to minimize infection risk and maximize therapeutic efficacy. To minimize potential problems and improve patient outcomes, clinicians handling these situations need to take a vigilant approach to monitoring and treatment, with a strong emphasis on thorough evaluations and attentive follow-up [11].

A study carried out by Zhang et al. revealed that glucocorticoid therapy was associated with a lower relapse-free survival rate. This suggests that sirolimus could be a promising therapeutic option for the treatment of ES and refractory or relapsed warm AIHA. According to the study, a sizable majority of patients reacted effectively to sirolimus, experiencing either a full or partial response with tolerable side effects such as infections and mucositis. Significantly, improved treatment outcomes were shown by individuals with greater sirolimus plasma trough concentrations, highlighting the significance of therapeutic drug monitoring in maximizing efficacy. These results highlight sirolimus as a beneficial substitute for patients who do not respond well to traditional glucocorticoid therapy, providing extended disease control and possibly lowering the relapse risk during extended follow-up periods [12].

A study by Fattizzo et al. assessed the safety and effectiveness of thrombopoietin receptor antagonists (TPO-RAs) in ES patients. Response rates of 80% at different time points were shown in the results, which showed encouraging results with TPO-RAs in ES. With response rates similar to those seen in primary ITP, the majority of patients showed notable increases in platelet counts. Remission without treatment was notable for a portion of patients, but recurrence rates highlighted how chronic and persistent ES is. Nevertheless, there were noticeable drawbacks to using TPO-RAs, such as a high rate of treatment-related adverse events, especially thrombotic events, which affected almost one-third of patients and were far more common than those seen in primary ITP populations. This emphasizes the necessity of cautious management techniques and close observation in order to minimize risks and maximize therapeutic benefits in this challenging and complex patient population [9].

A list of similar cases published in the literature is shown in Table 3.

**Table 3:** Review of literature outlining similar cases AIHA, autoimmune hemolytic anemia; DAT, direct antiglobulin test; DIC, disseminated intravascular coagulation; DVT, deep vein thrombosis; ES, Evans syndrome; FFP, fresh frozen plasma; HCT, hematocrit test; HGB, hemoglobin; HUS, hemolytic-uremic syndrome; PBS, peripheral blood smear; PCR, polymerase chain reaction; PEX, plasma exchange; PLT, platelet; PRP, platelet-rich plasma; TB, tuberculosis; TTP, thrombotic thrombocytopenic purpura

Author	Age/sex	Clinical features	Diagnosis	Treatment	Outcome
Angelopoulos et al. (2021) [1]	83 years old/female	Petechiae and diffuse ecchymosis over the limbs and body, including a 6- to 7-cm diameter ecchymosis in the umbilicus. Regular breathing patterns and a painless belly without a palpable spleen or liver.	A positive direct Coombs test result, a normal PBS, and a bone marrow examination are indicative of ES. Anemia (HGB 7.4 g/dl), low HCT (23.6%), RBC (2.36 K/μ), and thrombocytopenia (PLT 61,000/μl) were the initial lab results.	RBC concentrates and FFP transfusions; 120 mg/day intravenous methylprednisolone; and, in the event of a thrombocytopenia relapse, 1.5 mg/kg/day prednisolone.	Hematocrit increased to 33% in the first instance of improvement, although blood cell counts stayed the same. Following prednisolone treatment, the PLT count increased to 113 K/ml, indicating a remarkable recovery in the patient’s second hospitalization due to a thrombocytopenia relapse.
Dhakal et al. (2022) [7]	50 years old/female	Bleeding from the mouth and gums, bluish areas on the leg and trunk, weakness, excruciating back pain lasting seven days, and blood in the stool.	Low PLT count, hemoglobin, RBC count, and neutrophilic leukocytosis. Good direct Coombs test result. TTP and DIC were ruled out by differential diagnosis.	One pint of packed blood cells, three pints of PRP, intravenous steroids, vitamin B12, antibiotics, and Rituximab transfusions. Rituximab and steroid pulse treatment every week.	Gradual improvement in the hematological and clinical conditions. discharged on the 14th day. She was stable at the eight-week check-in.
Yang and Chen (2021) [10]	33 years old/female	Presented with left lower extremity swelling that was getting worse, erythema, warmth, soreness, and a 4 cm larger left leg than a right. Previous right immature ovarian teratoma treated with surgery and chemotherapy; history of ES; recent usage of low-dose prednisolone. A right atrial thrombus that extended into the inferior vena cava and right ventricle, as well as iliofemoral DVT, were discovered.	Primary antiphospholipid syndrome (lupus anticoagulant positive, decreased protein S level), with associated thrombotic events including iliofemoral DVT and right atrial thrombus.	Initially, 6,000 IU of subcutaneous enoxaparin sodium every 12 hours. Due to the right atrial mass’s inadequate anticoagulation, an open thrombectomy was done. Oral warfarin was part of the postoperative care for six months.	A lower mini-sternotomy was used to successfully remove the organized thrombus from the right atrium surgically. The left leg’s edema improved during the uncomplicated postoperative period. Prior to discharge, an echocardiogram revealed mild residual tricuspid valve incompetence. At the six-month follow-up, no new issues were noted.
Pan et al. (2022) [11]	69 years old/female	Fever and dyspnea for five days, along with overall weakness and poor appetite for more than a month. Both lung fields have large miliary nodules. Rapidly dropping oxygen saturation to the point where mechanical breathing and intubation are needed. Found to have serious TB in the lungs.	Acute respiratory failure, ES, DIC, thrombocytopenia, hepatic failure, renal insufficiency, and hypoalbuminemia are among the conditions associated with subacute hematogenous disseminated pulmonary TB. IgG PLT antibody and the direct Coombs test results are positive.	Recombinant human thrombopoietin, intravenous dexamethasone, antibiotics (sulbactam and cefoperazone), mechanical breathing, anti-tubercular treatment, and subsequently oral prednisone and levofloxacin.	Enhanced oxygenation, elevated hemoglobin level (to 80 g/L), and increased PLT count (to 63 × 10⁹/L). On November 8, 2021, the patient was extubated and then released with follow-up care in a stable condition.
Makharia et al. (2021) [13]	55 years old/male	Thrombocytopenia, anemia, lethargy, and generalized weakness for a month.	ES was identified by ruling out other illnesses and obtaining a positive DAT.	Packed RBC and PLT transfusions, oral prednisolone, pulse methylprednisolone, rituximab, and splenectomy. kept on preventative antibiotics and azathioprine.	After seven days, the PLT count rose to 25,000/mm³, and after a month, it reached 49,000/mm³. It improved to 8.3 g/dL for hemoglobin. Follow-up is still done on a regular basis.
Couri and Kandula (2020) [14]	60 years old/male	Sudden onset of hematuria and significant low back discomfort. The following conditions can occur: anemia, scleral icterus, low to normal PLT counts, moderate jaundice, and generalized peripheral edema. A history of DVT, body aches, sore throat, and recent ibuprofen use.	The patient had low ADAMTS-13 activity (39%), hemoglobin cast nephropathy, microangiopathy, antiplatelet antibodies (GPIIb/IIIa, GPIb/IX, and GPIa/IIa), and “warm” IgG and C3d antibodies.	High-dose corticosteroids (methylprednisolone 1 g), intermittent hemodialysis, PEX, prednisone 1 mg/kg for 30 days, and intravenous rituximab 100 mg every week for four weeks.	Decreased hemolysis, enhanced kidney function, and increased urine production. released from the hospital while receiving high-dose prednisone, ongoing rituximab, and sporadic hemodialysis. A bone marrow biopsy revealed little hypocellularity in the absence of notable abnormalities or tumors.
Demir et al. (2021) [15]	22 years old/male	Severe anemia (3.9 g/dL), fever, jaundice, weakness, and grade IV thrombocytopenia (86 × 10^9^/L). Notwithstanding a negative SARS-CoV-2 PCR result, thoracic CT scans indicated COVID-19 pneumonia. AIHA was indicated by the development of hemolysis markers and a positive direct Coombs test result (IgG 4+). An erythroblast biopsy revealed dysplastic alterations in the bone marrow.	ES, associated with SARS-CoV-2, characterized by combined AIHA and thrombocytopenia.	Despite a negative PCR result, the patient was initially treated with hydroxychloroquine, moxifloxacin, and favipiravir for suspected COVID-19. Began taking a proton pump inhibitor, folic acid, vitamin B12, and methylprednisolone. A daily erythrocyte suspension is necessary. Due to AIHA and persistent thrombocytopenia, plasmapheresis was started. IVIG treatment (1 g/kg/day) was administered after, improving the PLT count.	Hemoglobin stabilized at 8 g/dL without the need for erythrocyte suspension during plasmapheresis and IVIG treatment. The PLT count increased to 24 × 10^9^/L and kept going up. When the PLT count increased to >50 × 10^9^/L, enoxaparin treatment was restarted. Hemoglobin was 13 g/dL, PLTs were 210 × 10^9^/L, and the fast antibody test for SARS-CoV-2 was positive (IgM and IgG) at the time of discharge.

## Conclusions

ES is a chronic, recurrent illness with substantial diagnostic and treatment problems. It is characterized by AIHA and ITP. A comprehensive differential diagnosis is necessary to rule out conditions like SLE and lymphoproliferative disorders. Hematologic assessments, such as DAT, PBS, and CBC, are crucial. A swift response to corticosteroids in this case emphasizes the value of considering an autoimmune process while constructing differential diagnoses. The multifaceted medical background of this patient can further increase the pragmatic challenges in real clinical scenarios. Close patient monitoring and an individualized treatment plan are pivotal in achieving the aim of gaining remission and lowering the odds of relapse, thereby improving outcomes. First-line therapies usually consist of corticosteroids and IVIG, although rituximab and TPO-RAs are also available. Immunosuppressors such as sirolimus provide hope to patients who are not responding to conventional therapy. Vigilant management is required for complications such as thromboembolic events and serious infections. Despite advancements, recurrence rates for ES are high and the condition is still chronic; thus, achieving the best possible outcomes and enhancing quality of life requires prompt diagnosis, careful treatment, and extensive patient monitoring.
